# The Severity of Diabetic Retinopathy Is an Independent Factor for the Progression of Diabetic Nephropathy

**DOI:** 10.3390/jcm10010003

**Published:** 2020-12-22

**Authors:** Shi-Chue Hsing, Chia-Cheng Lee, Chin Lin, Jiann-Torng Chen, Yi-Hao Chen, Wen-Hui Fang

**Affiliations:** 1 Department of Internal Medicine, Tri-Service General Hospital, National Defense Medical Center, Taipei City 11490, Taiwan; lars0121@gmail.com; 2 Planning and Management Office, Tri-Service General Hospital, National Defense Medical Center, Taipei City 11490, Taiwan; clee112@ndmctsgh.edu.tw; 3 Division of Colorectal Surgery, Department of Surgery, Tri-Service General Hospital, National Defense Medical Center, Taipei City 11490, Taiwan; 4 Graduate Institute of Life Sciences, National Defense Medical Center, Taipei City 11490, Taiwan; xup6fup0629@gmail.com; 5 School of Medicine, National Defense Medical Center, Taipei City 11490, Taiwan; 6 School of Public Health, National Defense Medical Center, Taipei City 11490, Taiwan; 7 Department of Ophthalmology, Tri-Service General Hospital, National Defense Medical Center, Taipei City 11490, Taiwan; jt66chen@gmail.com (J.-T.C.); doc30879@mail.ndmctsgh.edu.tw (Y.-H.C.); 8 Department of Family and Community Medicine, Tri-Service General Hospital, National Defense Medical Center, Taipei City 11490, Taiwan

**Keywords:** diabetic retinopathy, type 2 diabetes, progression, end-stage renal disease, chronic kidney disease

## Abstract

(1) Background: It has rarely been studied whether the severity of diabetic retinopathy (DR) could influence renal disease progression in end-stage renal disease (ESRD) and chronic kidney disease (CKD) in patients with type 2 diabetes. The aim of this study was to evaluate renal disease progression in ESRD and CKD according to DR severity in patients with type 2 diabetes. (2) Methods: We included 1329 patients and divided the cohort into two end-points. The first was to trace the incidence of ESRD in all enrolled participants and the other was to follow their progression to CKD. (3) Results: Significantly higher crude hazard ratios (HRs) of ESRD incidence in all enrolled participants were noted, and this ratio increased in a stepwise fashion. However, after adjustment, DR severity was not associated with ESRD events. Therefore, a subgroup of 841 patients without CKD was enrolled to track their progression to CKD. Compared with no diabetic retinopathy, the progression of CKD increased in a stepwise fashion, from mild nonproliferative diabetic retinopathy (NPDR) to moderate NPDR, to severe NPDR and to proliferative diabetic retinopathy (PDR), both in the crude and adjusted models. (4) Conclusions: The severity of retinopathy appeared to be associated with renal lesions and the development of CKD. Our findings suggest that the severity of DR is a risk factor for progression to CKD. Therefore, diabetic retinopathy is useful for prognosticating the clinical course of diabetic kidney disease.

## 1. Introduction

The major microvascular complications of diabetes mellitus (DM) include retinopathy, nephropathy, and neuropathy. Diabetic retinopathy progresses stepwise from milder stages of nonproliferative diabetic retinopathy (NPDR) to more advanced vision-threatening levels, including proliferative diabetic retinopathy (PDR) and diabetic macular edema (DME). Diabetic retinopathy (DR) and diabetic nephropathy (DN) share common risk factors, such as poor glycemic control and systolic hypertension. Both have similar pathological abnormalities within the glomerular and retinal vessels [1,2,3].

Longitudinal studies have investigated whether the presence of retinopathy is associated with chronic kidney disease (CKD) progression among all patients with type 2 diabetes and chronic kidney disease [4,5,6,7]. However, the association between the severity of DR and renal disease progression among patients with type 2 diabetes has rarely been studied and is unclear. A Taiwan case-control study among patients with type 2 diabetes revealed that PDR is significantly associated with CKD progression but NPDR is not [8]. A Japanese cohort of biopsy-proven diabetic kidney disease among patients with type 2 diabetes showed the risk for end-stage renal disease (ESRD) increased in a stepwise fashion, from mild NPDR to moderate NPDR, to severe NPDR, to PDR [9]. A Korean retrospective study among patients with type 2 diabetes showed that NPDR and PDR were associated with CKD progression [6]. All of these studies revealed PDR is associated with DN progression. However, the association with NPDR is relatively unclear. According to the Japanese cohort study, distinguishing among the different NPDR stages may help us to clarify this issue.

We postulated that DR and DN share similar mechanisms, resulting in the concurrent progression of both complications. The aim of this study was to evaluate the renal disease progression (ESRD and CKD) in patients with type 2 diabetes and with/without diabetic kidney disease according to DR severity (from mild NPDR to moderate NPDR, to severe NPDR, to PDR).

## 2. Materials and Methods

### 2.1. Population

**Figure 1:**
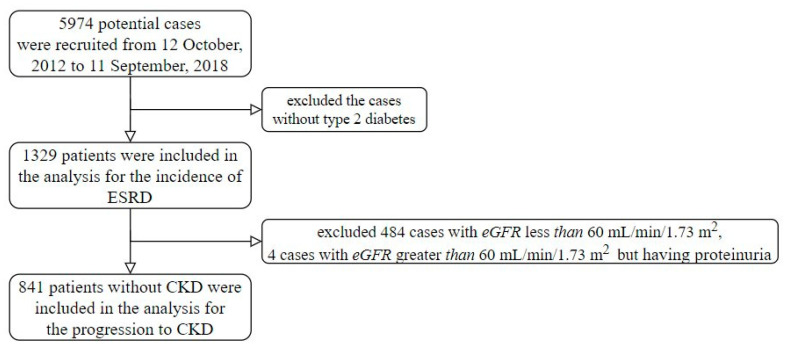
Recruitment process flow chart.

The Tri-Service General Hospital, Taipei, Taiwan, provided their research data from 12 October 2012 to 11 September 2018. Research ethics approval was given by the institutional review board to collect data without individual consent (IRB NO 1-107-05-047). Any patients with diabetes and more than one fundus color photograph session were included in this study. The start of follow-up was defined as the first fundus color photograph session. There were 5974 potential cases included in this study, but we excluded the cases without type 2 diabetes. The definition of diabetes was one of the following conditions: (1) At least two International Classification of Diseases (ICDs) of type 2 diabetes (ICD9: 250 or ICD10: E11) from half a year ago to the start time and received oral hypoglycemic agents or insulin treatments, (2) at least 2 records of more than or equal to 126 mg/dL of fasting glucose from half a year ago to the start time, and/or (3) at least 2 records of more than or equal to 6.5% of HbA1C from half a year ago to the start time. Moreover, patients were excluded if they had one of the following conditions: (1) At least one ICD of end-stage renal disease (ESRD), which is ICD9:585.6 or ICD10:N18.6, (2) lack of serum creatinine measurement within 3 days of the start time, and/or (3) at least 2 records of serum creatinine measurements after the start time. Finally, there were 1329 patients included in this study (Figure 1). We divided the cohort into two end-points. The first was traced to the incidence of ESRD in all enrolled participants and the other was people without CKD at the time of enrollment, 841 people, followed until their progression to CKD.

### 2.2. Measurements and Variables

DR was defined according to the following retinal microvascular lesions: Microaneurysms, hard exudates, intraretinal hemorrhages, venous beading, or prominent intraretinal microvascular abnormality, retinal or optic disk neovascularization, vitreous hemorrhage, or preretinal hemorrhage. DR was graded on the International Clinical Diabetic Retinopathy Disease Severity Scale [10] as follows: (0) No apparent retinopathy; (1) mild NPDR: Microaneurysms only; (2) moderate NPDR: Any microaneurysms, dot and blot hemorrhages, hard exudates or cotton wool spots, but less than severe NPDR; (3) severe NPDR: Intraretinal hemorrhages (≥20 in each of four quadrants), definite venous beading (in two quadrants), or intraretinal microvascular abnormalities ((in one quadrant), but no signs of proliferative retinopathy; (4) PDR: One or more of neovascularization, vitreous, or preretinal hemorrhages.

The stages of CKD were determined according to the Kidney Disease Outcomes Quality Initiative guidelines using the eGFR as calculated by the Chronic Kidney Disease Epidemiology Collaboration equation [11]. The various CKD stages included the following: Stage 1, eGFR ≥ 90 mL/min/1.73 m^2^ and the presence of any kidney damage; stage 2, eGFR = 60–89 mL/min/1.73 m^2^ with any kidney damage; stage 3, eGFR = 30–59 mL/min/1.73 m^2^; stage 4, eGFR = 15–29 mL/min/1.73 m^2^; stage 5, eGFR < 15 mL/min/1.73 m^2^ [12].

We collected other laboratory records within 3 days: Total cholesterol, low-density lipoprotein (LDL), High-density lipoprotein (HDL), triglyceride, alanine aminotransferase (ALT), creatinine, hemoglobin, and white blood cell (WBC) and platelet counts. The missing rate of the above variables in this study was less than 30%. We used the multiple imputations method to impute the missing values. The other demographic characteristics and comorbidities were collected from electronic health records. The basic characteristics included sex, age, height, weight, systolic blood pressure (SBP), and diastolic blood pressure (DBP). The definition of comorbidities was based on the corresponding International Classification of Disease Ninth Revision and Tenth Revision. The comorbidities of hypertension, ischemic heart disease, stroke, and diabetic neuropathy were included in this study. An ESKD event was defined as the initiation of any hemodialysis, peritoneal dialysis, renal transplantation, or death from uremia. An eGFR decline to less than 60 mL/min/1.73 m^2^ was defined as progression to CKD.

### 2.3. Deep Learning Model for Grading Diabetic Retinopathy

The evaluations for grading diabetic retinopathy were conducted by the artificial algorithm that we developed previously and based on convolutional neural network. The fundus color photograph session were provided by the Kaggle coding website [13], which contained over 35,126 images corresponding scales of diabetic retinopathy. The model architecture was based on a 50-layer SE-ResNeXt, [14] which won the ImageNet Large-Scale Visual Recognition Challenge (ILSVRC) in 2017. We revised the number of hidden neurons in last fully connected layer from 1000 to 5 for fitting our task. All initial weights were based on the SE-ResNeXt is pretrained by ImageNet except the last layer. Due to significantly uneven distribution of each grade of diabetic retinopathy, an oversampling process was implemented to ensure that rare samples were adequately recognized [15,16]. The settings for the training model were as follows: (1) Adam optimizer with standard parameters (β_1_ = 0.9 and β_2_ = 0.999) and a batch size of 32 for optimization; (2) learning rate was set at 0.001; and (3) a weight decay of 10^−4^ [17]. The 100th epoch model was used as the final model. The software package MXNet version 1.3.0 was implemented to our deep learning model. The public score and private score of our deep learning model in a test set involving 53,576 images were 0.837 and 0.841, respectively, which were the seventh and third place in the leaderboard. This model was used to apply in our database to evaluate the grade of diabetic retinopathy in each fundus color photograph session.

The final DR severity was confirmed by ophthalmologist and was determined by the result of the more severely affected eye.

### 2.4. Statistical Analysis and Model Performance Assessment

We presented the characteristics as the means and standard deviations, numbers of patients, or percentages, where appropriate. They were compared using either analysis of variance or chi-square tests, as appropriate. We used a significance level of *p* < 0.05 throughout the analysis. The statistical analysis was carried out using the software environment R version 3.4.3.

The primary analysis was to evaluate the effects of different grades and the progression of diabetic retinopathy. We used Kaplan–Meier curves to present the progression difference between participants with HbA1c and each initial grade. All variables were evaluated for their association with diabetic retinopathy progression using univariate Cox proportional hazard models. A multivariable Cox proportional hazard model was used to adjust the potential confounding factors, and the selection of the adjusted variables was based on the significance of the univariate analysis.

The secondary analyses were performed by stratified analysis. We only presented the analysis of the most significant type of initial grade of diabetic retinopathy progression. Because the initial conditions of different patients were variable in real clinical practice, we tried to use the initial grade of diabetic retinopathy and the baseline HbA1c as the stratified variables. The interaction analysis was based on a Cox proportional hazard model, and the adjusted variables were the same as the primary analysis.

## 3. Results

Table 1 presents the basic characteristics of the patients with the grade of diabetic nephropathy and Table 2 presents the basic characteristics of the patients by the DR grade. The average age of the 1329 patients was 63.28 ± 12.75 years old, 52.9% were male, and 484 patients had CKD at the time of enrollment. According to the International Clinical Diabetic Retinopathy Disease Severity Scale, 503 patients were classified as no diabetic retinopathy, 243 patients as mild NPDR, 378 patients as moderate NPDR, 170 patients as severe NPDR, and 35 patients as PDR.

A total of 1329 patients were included in this study. We divided them into two end-points. The first was for the incidence of ESRD in all enrolled participants. The other was for people without CKD at the time of enrollment, 841 people in total, followed for their progression to CKD. A significantly higher crude HR of ESRD incidence in all enrolled participants was noted. The disease progressed in a stepwise fashion, from mild NPDR to moderate NPDR, to severe NPDR, to PDR. The crude HR of progression to ESRD events were 2.35 (1.01–5.43) in mild NPDR, 5.96 (2.99–11.89) in moderate NPDR, 2.79 (1.16–6.75) in severe NPDR, and 6.98 (2.22–21.94) in PDR compared to the non-DR group. However, after adjustment (baseline eGFR, age, BMI, sex), DR severity was not associated with ESRD events. Table 3 presents the risk of progression to ESRD through the end of the study.

A total of 845 patients’ eGFR was greater than 60 mL/min/1.73 m^2^. However, only four had proteinuria. Therefore, 841 patients without CKD were enrolled for an evaluation of their progression to CKD. Table 4 presents the characteristics of the patients without CKD different initial grades of diabetic retinopathy. Their DR severity was related to a younger age, poor glycemic control (HbA1c), and a higher systolic blood pressure, which is similar to that reported in a previous study [4,5,6]. Baseline eGFR, sex, and BMI did not significantly differ between each group.

Compared with no diabetic retinopathy, the HR for the progression of CKD increased in a stepwise fashion, from mild NPDR to moderate NPDR, to severe NPDR, to PDR, both in the crude and adjusted model ((adjustment for baseline eGFR, age, BMI, and sex). The crude HRs of progression to CKD events were 3.46 (95% CI 0.92 to 12.98) for patients with mild NPDR, 8.75 (95% CI 2.72 to 27.92) for patients with moderate NPDR, 5.73 (95% CI 1.63 to 20.04) for patients with severe NPDR, and 14.21 (95% CI 1.55 to 130.67) for patients with PDR. The adjusted HRs of progression to CKD events were 3.38 (95% CI 0.90 to 12.71) for patients with mild NPDR, 7.88 (95% CI 2.43 to 25.57) for patients with moderate NPDR, 5.08 (95% CI 1.35 to 19.04) for patients with severe NPDR, and 11.43 (95% CI 1.22 to 107.12) for patients with PDR. Table 5 presents the risk of progression to CKD by the end of the study among patients without CKD at the start of the study.

**Figure 2:**
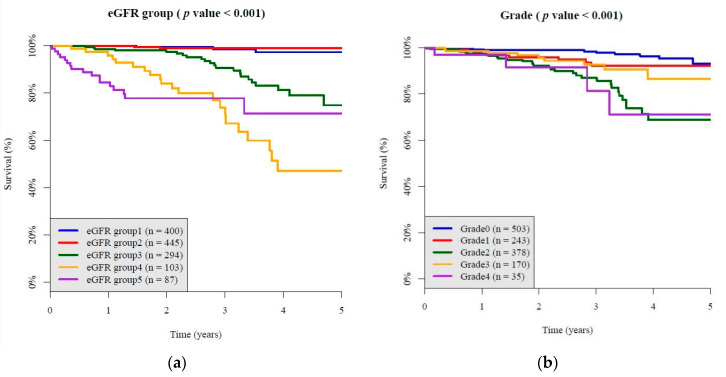
The Kaplan–Meier survival curve showed progression of diabetic retinopathy comparison in each grade and different tertiles of baseline HbA1C. (**a**) A stepwise fashion of ESRD incidence in all enrolled participants with different initial grade of diabetic nephropathy; (**b**) a stepwise fashion of ESRD incidence in all enrolled participants, from mild NPDR to moderate NPDR, to severe NPDR, to PDR.

**Figure 3:**
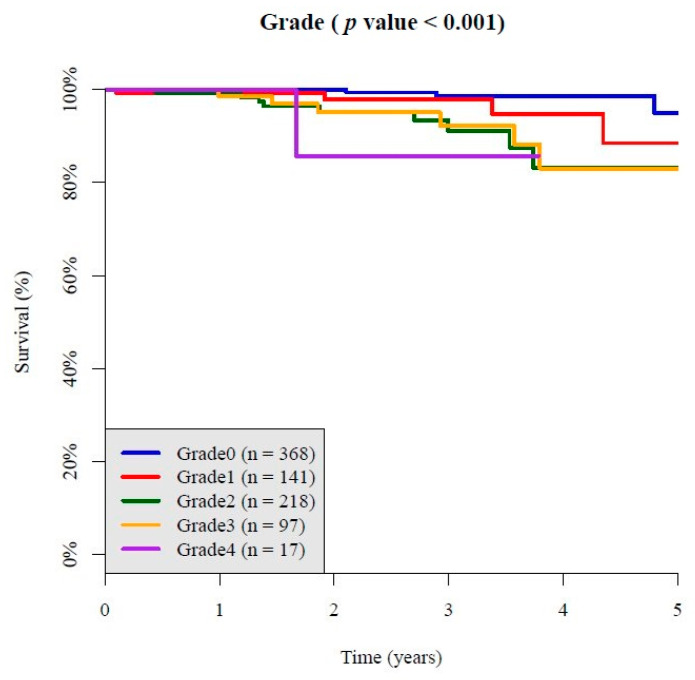
The Kaplan–Meier survival curve showed a stepwise fashion of CKD incidence from mild NPDR to moderate NPDR, to severe NPDR, to PDR.

As shown in Figure 2 and Figure 3, the Kaplan–Meier survival curve showed that initial grades of diabetic DR was related to ESRD incidence and CKD incidence. The higher the severity scale of diabetic retinopathy went up, the more ESRD incidence and CKD incidence became (Figure 2b: *p* < 0.001; Figure 3: *p* < 0.001). The Kaplan–Meier survival curve in each subgroup was showed in Supplementary Figure (Figures S1–S3), which also showed DR grade was related to the incidence of ESRD and progression to CKD.

## 4. Discussion

In patients with diabetes and without CKD initially, we found the progression of CKD proceeded in a stepwise fashion, from mild NPDR to moderate NPDR, to severe NPDR, to PDR, both in the crude and adjusted model (adjustment for baseline eGFR, age, BMI, and sex). This finding suggested that the DR severity is an independent factor related to renal functional decline in non-CKD patients with diabetes.

Previous studies demonstrated that the presence of DR is associated with an increased risk of end-stage renal disease [4], albuminuria progression [6], and accelerated eGFR decline [5,6]. DR severity may be independently associated with CKD progression. A meta-analysis revealed that PDR is more specific than NPDR in screening for DN and that severe retinopathy is associated with later CKD stages [18]. A Korean population-based retrospective study showed that NPDR has a 2.9 times and PDR has a 16.6 times higher risk for CKD progression than the no DR group after adjustment [6]. However, a multicenter case–control study in Taiwan revealed that PDR is significantly associated with CKD progression after adjustments for baseline eGFR, hypertension, HbA1c, and BMI, but NPDR is not [8]. A longitudinal Japanese cohort of patients with type 2 diabetes with biopsy-proven diabetic kidney disease showed the risk for ESRD increased in a stepwise fashion after adjusting from mild NPDR to moderate NPDR, to severe NPDR, to PDR [9]. The hazard ratios (HRs) of progression to ESRD events were 1.96 (95% CI 0.62 to 6.17) in mild NPDR, 3.10 (95% CI 1.45 to 6.65) in moderate NPDR, 3.03 (95% CI 1.44 to 6.37) in severe NPDR, and 3.43 (95% CI 1.68 to 7.03) in PDR, respectively, compared to the non-DR group after adjusting for the known risk factors of ESKD.

This study revealed DR severity might be associated with a faster renal functional decline. PDR was associated with DN progression. NPDR was associated with DN progression in some but not all studies. In some studies, the renal outcome (such as the albumin excretion rate, nephropathy presentation rate, risk for ESRD) was significantly higher among subjects with either PDR or severe NPDR relative to mild NPDR [9,19,20]. This suggested that severe NPDR might have more influence on DN than mild NPDR and that worsening retinopathy is associated with later CKD stages. Distinguishing mild, moderate and severe NPDR might help us to clarify the effect of DR severity on renal disease progression. Racial and genetic factors also affect the association between DN and DR in type 2 diabetes [21,22].

Thus, in this Taiwan population-based study, we evaluated the risk for ESKD and CKD progression according to the severity of diabetic retinopathy from mild NPDR to moderate NPDR, to severe NPDR, to PDR. In our study, we found DR severity was independently associated with progression to CKD. The ESRD events among patients with diabetes were significantly associated with DR severity in a stepwise fashion. However, the association was not maintained in the adjusted model. Our study cohort included all CKD stages. A previous study found that DR is significantly correlated with CKD progression in later CKD stages [8]. This may be because the association between DR severity and ESRD events was weaker than for the baseline CKD stage. Therefore, after adjustment for baseline eGFR, no significant association between DR severity and ESRD events was found. Therefore, we analyzed patients without CKD initially to follow their progression to CKD. In patients with diabetes without CKD initially, we found the progression of CKD increased in a stepwise fashion, from mild NPDR to moderate NPDR, to severe NPDR, to PDR, both in the crude and adjusted model ((adjusted for baseline eGFR, age, BMI, and sex).

There were significant differences in DR severity, age, uric acid, albumin and more hypertension events than in other studies [8,23,24]. Patients with DR had a worse baseline eGFR, worse glycemic control (higher HbA1c) and higher uric acid. In the baseline data, we observed that the DR group was younger than the non-DR group in our population. Other studies have also found that patients with DR are younger. The reason for this is unclear; however, patients with diabetes clearly should be evaluated for DR as early as possible.

Our study had a higher prevalence of NPDR but less PDR compared to previous studies (NPDR = 59.5%, PDR = 2.6% vs. NPDR 23.7%, PDR 12.7% [8]; and vs. NPDR = 45.68%, PDR = 30.6% [9]; and NPDR = 24.1%, PDR= 12.7% [6], respectively). Better renal function was also noted in our study. In spite of the absence of information about the duration of DM in this study, the above findings suggest our study group is in an earlier stage of DM compared to a previous similar studies.

The prevention of diabetic nephropathy is treatment of its known risk factors. Intensive blood glucose control, intensive blood pressure control, renin-angiotensin system blockade with angiotensin-converting enzyme inhibitors (ACEi) or angiotensin receptor blockers (ARBs), dietary protein restriction, and lipid-lowering therapy contribute to diminishing the risk of diabetic nephropathy [25,26,27]. Considering that DR severity is independently associated with progression to CKD and our study group is in an earlier stage of DM, follow-up and management of retinal disorders in patients with diabetes is important for prevention of the progression to CKD. Early initiation of renoprotective therapy in patients with DR without/with nephropathy might be important. Additional studies on the influence of DN prevention management on renal outcomes among patients with DR without nephropathy might be important.

The strengths of our study are the use of a longitudinal design rather than a cross-sectional design, including all CKD stages, and good validation of the grading of diabetic retinopathy. Our study has several limitations. First, the data in this cohort study were collected retrospectively from a single center. Sampling bias and selection bias is inevitable. The results could not be used to establish a cause-effect relationship. However, patients who were newly diagnosed with type 2 DM were routinely transferred to receive a fundus examination in our hospital. This study was sponsored by the national health insurance, and nearly 99.9% of the Taiwanese population is under National Health Insurance coverage [28]. Second, the DM duration prior to enrollment was not recorded in our study. Disease duration is one of the main risk factors of CKD prevalence and DR prevalence. However, the baseline eGFR and the distribution of DR severity indicated our study group was in the early disease course of DM. Nevertheless, the duration of diabetes may still be a confounder. Finally, this analysis was performed in Taiwanese patients with type 2 DM, and the results need to be confirmed in other populations.

## 5. Conclusions

In conclusion, DR severity was a prognostic factor for progression to CKD in patients with type 2 DM. Therefore, clinicians must evaluate the DR severity at the first visit. DR severity may be a powerful tool in predicting the clinical course of diabetic kidney disease in patients with type 2 diabetes. Routine follow-up and management of retinal disorders in all patients with type 2 diabetes would be important. Early initiation of renoprotective therapy in patients with DR may also be a future direction in the management of these patients.

## Figures and Tables

**Table 1:** The characteristics of patients with different initial grades of diabetic nephropathy.

	Initial Grades of Diabetic Nephropathy					
	Stage 1 (*n* = 400)	Stage 2 (*n* = 445)	Stage 3 (*n* = 294)	Stage 4 (*n* = 103)	Stage 5 (*n* = 87)	*p*-Value #
Basic characteristics						
DR severity						<0.001
No DR	175 (43.8%)	193 (43.4%)	93 (31.6%)	21 (20.4%)	21 (24.1%)	
Mild NPDR	73 (18.2%)	69 (15.5%)	56 (19.0%)	25 (24.3%)	20 (23.0%)	
Moderate NPDR	106 (26.5%)	114 (25.6%)	94 (32.0%)	36 (35.0%)	28 (32.2%)	
Severe NPDR	39 (9.8%)	59 (13.3%)	42 (14.3%)	16 (15.5%)	14 (16.1%)	
PDR	7 (1.8%)	10 (2.2%)	9 (3.1%)	5 (4.9%)	4 (4.6%)	
Gender						0.222
Female	171 (42.8%)	222 (49.9%)	147 (50.0%)	46 (44.7%)	40 (46.0%)	
Male	229 (57.2%)	223 (50.1%)	147 (50.0%)	57 (55.3%)	47 (54.0%)	
Age (years)	57.58 ± 12.88	65.92 ± 11.29	67.12 ± 12.45	65.01 ± 11.71	60.91 ± 12.05	<0.001
Height (cm)	162.48 ± 9.05	161.84 ± 8.77	161.94 ± 8.60	162.24 ± 8.59	163.25 ± 8.73	0.625
Weight (kg)	69.05 ± 15.04	66.27 ± 13.09	67.62 ± 14.27	67.20 ± 12.73	67.24 ± 13.01	0.076
Body mass index	26.06 ± 4.97	25.20 ± 4.01	25.74 ± 4.97	25.47 ± 3.96	25.21 ± 4.31	0.074
SBP (mmHg)	138.75 ± 21.51	141.20 ± 21.45	141.94 ± 22.83	138.43 ± 23.45	141.99 ± 22.58	0.235
DBP (mmHg)	80.56 ± 12.52	79.36 ± 12.55	78.20 ± 12.59	77.19 ± 11.43	77.95 ± 13.46	0.039
Comorbidity						
Hypertension	120 (30.0%)	166 (37.3%)	137 (46.6%)	53 (51.5%)	48 (55.2%)	<0.001
lipidemia	128 (32.0%)	132 (29.7%)	87 (29.6%)	33 (32.0%)	30 (34.5%)	0.850
Ischemic heart disease	81 (20.2%)	95 (21.3%)	77 (26.2%)	25 (24.3%)	33 (37.9%)	0.005
Heart failure	41 (10.2%)	40 (9.0%)	31 (10.5%)	8 (7.8%)	11 (12.6%)	0.760
COPD	7 (1.8%)	7 (1.6%)	12 (4.1%)	4 (3.9%)	1 (1.1%)	0.128
Stroke	66 (13.1%)	25 (10.3%)	52 (13.8%)	23 (13.5%)	5 (14.3%)	0.734
Diabetic neuropathy	38 (9.5%)	35 (7.9%)	23 (7.8%)	12 (11.7%)	7 (8.0%)	0.703
Laboratory test						
HbA1c (%)	8.21 ± 2.07	7.80 ± 1.85	8.19 ± 2.10	7.78 ± 1.78	7.95 ± 2.05	0.014
Last Glu AC (mg/dL)	149.85 ± 56.80	145.87 ± 58.83	149.61 ± 58.06	150.43 ± 68.97	140.93 ± 51.79	0.617
Triglyceride (mg/dL)	151.19 ± 126.72	153.36 ± 139.92	160.72 ± 93.66	175.69 ± 89.07	172.44 ± 119.66	0.251
Total Cholesterol (mg/dL)	171.80 ± 39.92	170.43 ± 38.57	169.38 ± 46.76	179.24 ± 52.56	174.89 ± 61.73	0.321
LDL Cholesterol (mg/dL)	102.95 ± 33.15	100.64 ± 31.83	98.45 ± 36.81	98.70 ± 37.67	97.39 ± 47.30	0.416
HDL Cholesterol (mg/dL)	47.44 ± 13.33	46.77 ± 13.41	45.33 ± 11.83	45.00 ± 12.62	46.05 ± 13.61	0.192
Creatinine (mg/dL)	0.69 ± 0.14	0.93 ± 0.16	1.43 ± 0.30	2.83 ± 0.68	7.26 ± 2.81	<0.001
Uric Acid (mg/dL)	5.50 ± 1.47	5.85 ± 1.58	6.64 ± 1.93	7.24 ± 1.87	7.24 ± 2.51	<0.001
Hemoglobin (g/dL)	13.24 ± 1.76	13.03 ± 1.79	12.17 ± 1.88	10.62 ± 1.88	10.21 ± 1.51	<0.001
Albumin (g/dL)	3.88 ± 0.61	3.89 ± 0.57	3.79 ± 0.57	3.63 ± 0.57	3.47 ± 0.60	<0.001
Result						
ESRD event	4 (1.0%)	3 (0.7%)	22 (7.5%)	23 (22.3%)	17 (19.5%)	<0.001
Time to ESRD (years)	1.90 ± 1.53	2.05 ± 1.60	2.10 ± 1.53	1.83 ± 1.49	1.68 ± 1.29	0.080

#: Testing by Fisher’s exact test, Wilcoxon test, or Kruskal–Wallis test, respectively. Significance level of *p* < 0.05 throughout the analysis. DR = diabetic retinopathy, NPDR = nonproliferative diabetic retinopathy, PDR = proliferative diabetic retinopathy, SBP = Systolic blood pressure, DBP = Diastolic blood pressure, COPD = chronic obstructive pulmonary disease, HbA1c = Glycated hemoglobin, Glu AC = Glucose before a meal, LDL = Low-density lipoprotein, HDL = High-density lipoprotein, ESRD = end-stage renal disease.

**Table 2:** The characteristics of patients with different initial grades of diabetic retinopathy.

	Initial Grades of Diabetic Retinopathy					
	No DR (*n* = 503)	Mild NPDR (*n* = 243)	Moderate NPDR (*n* = 378)	Severe NPDR (*n* = 170)	PDR (*n* = 35)	*p*-Value #
Basic characteristics						
Body mass index	25.47 ± 4.27	25.89 ± 5.24	25.27 ± 4.28	26.18 ± 4.97	26.19 ± 4.22	0.152
SBP (mmHg)	138.85 ± 20.27	142.08 ± 22.72	141.37 ± 22.84	139.75 ± 22.69	146.14 ± 27.50	0.127
DBP (mmHg)	78.89 ± 12.26	78.33 ± 11.62	79.90 ± 13.04	79.54 ± 13.01	80.57 ± 15.44	0.527
Comorbidity						
lipidemia	144 (28.6%)	71 (29.2%)	120 (31.7%)	58 (34.1%)	17 (48.6%)	0.107
Ischemic heart disease	116 (23.1%)	56 (23.0%)	87 (23.0%)	47 (27.6%)	5 (14.3%)	0.491
Heart failure	49 (9.7%)	20 (8.2%)	41 (10.8%)	20 (11.8%)	1 (2.9%)	0.466
COPD	18 (3.6%)	5 (2.1%)	4 (1.1%)	2 (1.2%)	2 (5.7%)	0.051
Stroke	54 (13.5%)	48 (10.8%)	42 (14.3%)	18 (17.5%)	9 (10.3%)	0.305
Diabetic neuropathy	29 (5.8%)	26 (10.7%)	39 (10.3%)	15 (8.8%)	6 (17.1%)	0.018
Laboratory test						
HbA1c (%)	7.69 ± 1.89	8.03 ± 1.86	8.29 ± 2.01	8.39 ± 2.27	7.88 ± 1.91	<0.001
Last Glu AC (mg/dL)	143.29 ± 56.77	147.54 ± 55.95	151.82 ± 58.95	154.43 ± 65.91	143.43 ± 52.26	0.127
Triglyceride (mg/dL)	151.11 ± 111.56	155.49 ± 91.32	161.08 ± 129.90	170.54 ± 167.13	154.26 ± 106.63	0.444
Total Cholesterol (mg/dL)	170.58 ± 40.67	170.15 ± 45.89	172.11 ± 43.82	174.39 ± 48.75	176.74 ± 50.41	0.780
LDL Cholesterol (mg/dL)	99.70 ± 32.53	101.10 ± 37.07	99.40 ± 34.29	102.86 ± 38.61	107.66 ± 43.68	0.568
HDL Cholesterol (mg/dL)	47.31 ± 13.67	46.10 ± 12.27	45.98 ± 12.74	45.12 ± 12.60	48.71 ± 13.01	0.223
Creatinine (mg/dL)	1.28 ± 1.52	1.71 ± 2.09	1.63 ± 1.78	1.69 ± 1.68	2.15 ± 2.93	0.001
Uric Acid (mg/dL)	5.80 ± 1.67	6.16 ± 1.79	6.35 ± 2.04	6.33 ± 1.77	6.80 ± 1.55	<0.001
Hemoglobin (g/dL)	12.96 ± 1.85	12.53 ± 1.99	12.20 ± 2.12	12.23 ± 2.10	11.38 ± 1.94	<0.001
Albumin (g/dL)	3.89 ± 0.54	3.82 ± 0.57	3.77 ± 0.64	3.74 ± 0.63	3.66 ± 0.64	0.004
Result						
ESRD event	11 (2.2%)	11 (4.5%)	34 (9.0%)	9 (5.3%)	4 (11.4%)	<0.001
Time to ESRD (years)	2.22 ± 1.65	2.01 ± 1.50	1.60 ± 1.36	2.06 ± 1.51	1.82 ± 1.53	<0.001

#: Testing by Fisher’s exact test, Wilcoxon test, or Kruskal–Wallis test, respectively. Significance level of *p* < 0.05 throughout the analysis. SBP = Systolic blood pressure, DBP = Diastolic blood pressure, COPD = chronic obstructive pulmonary disease, HbA1c = Glycated hemoglobin, Glu AC = Glucose before a meal, LDL = Low-density lipoprotein, HDL = High-density lipoprotein, ESRD = end-stage renal disease.

**Table 3:** The risk of progression to ESRD before the end of the study.

	Crude-HR (95% CI)	*p*-Value	Adj-HR (95% CI)	*p*-Value #
Initial DR grade		<0.001		0.103
No DR	1.00		1.00	
Mild NPDR	2.35 (1.01–5.43)	0.046	1.51 (0.63–3.62)	0.355
Moderate NPDR	5.96 (2.99–11.89)	<0.001	2.63 (1.25–5.50)	0.010
Severe NPDR	2.79 (1.16–6.75)	0.023	1.58 (0.62–3.99)	0.335
PDR	6.98 (2.22–21.94)	0.001	1.87 (0.56–6.19)	0.308
Initial CKD grade		<0.001		<0.001
1	1.00		1.00	
2	0.42 (0.08–2.31)	0.321	0.67 (0.12–3.69)	0.642
3	8.27 (2.67–25.62)	<0.001	13.87 (4.42–43.54)	<0.001
4	27.37 (8.83–84.86)	<0.001	39.10 (12.54–121.88)	<0.001
5	30.27 (9.54–96.09)	<0.001	32.87 (10.36–104.21)	<0.001
Gender		0.252		0.956
Female	1.00		1.00	
Male	1.33 (0.81–2.19)	0.252	1.01 (0.61–1.67)	0.956
age	0.62 (0.51–0.76)	<0.001	0.55 (0.44–0.70)	<0.001
height	1.18 (0.92–1.51)	0.184	0.96 (0.71–1.30)	0.804
weight	0.95 (0.74–1.21)	0.669	0.88 (0.50–1.57)	0.676
Body mass index	0.86 (0.67–1.11)	0.254	0.85 (0.65–1.11)	0.230
SBP	0.91 (0.72–1.15)	0.439	0.91 (0.73–1.12)	0.359
DBP	0.98 (0.77–1.25)	0.877	0.88 (0.68–1.13)	0.311
Comorbidity				
Hypertension	2.68 (1.62–4.41)	<0.001	1.59 (0.95–2.66)	0.075
lipidemia	1.33 (0.82–2.18)	0.249	1.16 (0.70–1.92)	0.568
Ischemic heart disease	2.10 (1.29–3.41)	0.003	1.79 (1.08–2.97)	0.025
Stroke	1.44 (0.73–2.82)	0.293	1.46 (0.73–2.92)	0.287
Diabetic neuropathy	1.71 (0.89–3.28)	0.106	1.35 (0.67–2.70)	0.401
Heart failure	2.18 (1.19–4.01)	0.012	1.91 (1.01–3.60)	0.047
Laboratory test				
HbA1c	1.07 (0.86–1.33)	0.567	1.00 (0.79–1.27)	0.995
Glu AC	1.06 (0.87–1.31)	0.555	0.90 (0.76–1.08)	0.266
triglyceride	1.01 (0.95–1.06)	0.784	0.97 (0.91–1.03)	0.343
total Cholesterol	1.12 (0.98–1.29)	0.099	1.15 (0.94–1.41)	0.174
LDL Cholesterol	1.51 (1.26–1.82)	<0.001	1.23 (1.02–1.50)	0.033
HDL Cholesterol	0.95 (0.74–1.21)	0.653	0.83 (0.65–1.05)	0.119
Creatinine	1.54 (1.38–1.72)	<0.001	0.76 (0.52–1.12)	0.163
Hemoglobin	0.38 (0.30–0.49)	<0.001	0.75 (0.56–1.00)	0.049

#: All results of Adj-HR were adjusted by sex, age, BMI, eGFR. Significance level of *p* < 0.05 throughout the analysis. Crude-HR = Crude hazard ratios, Adj-HR = Adjusted hazard ratios, CI = Confidence interval, DR = diabetic retinopathy, NPDR = nonproliferative diabetic retinopathy, PDR = proliferative diabetic retinopathy, CKD = chronic kidney disease, SBP = Systolic blood pressure, DBP= Diastolic blood pressure, HbA1c = Glycated hemoglobin, Glu AC = Glucose before a meal, LDL = Low-density lipoprotein, HDL = High-density lipoprotein.

**Table 4:** The characteristics of non-CKD group patients at enrollment with different initial grades of diabetic retinopathy.

	Initial Grades of Diabetic Retinopathy					
	No DR (*n* = 368)	Mild NPDR (*n* = 141)	Moderate NPDR (*n* = 218)	Severe NPDR (*n* = 97)	PDR (*n* = 17)	*p*-Value #
Basic characteristics						
Gender						0.393
Female	182 (49.5%)	64 (45.4%)	94 (43.1%)	41 (42.3%)	10 (58.8%)	
Male	186 (50.5%)	77 (54.6%)	124 (56.9%)	56 (57.7%)	7 (41.2%)	
Age (years)	64.06 ± 13.90	61.69 ± 13.41	60.54 ± 10.28	58.58 ± 11.67	58.33 ± 10.10	<0.001#
Height (cm)	161.63 ± 8.85	162.10 ± 9.88	162.42 ± 8.37	163.57 ± 8.82	161.79 ± 9.63	0.298#
Weight (kg)	66.80 ± 13.83	68.17 ± 13.78	67.42 ± 13.71	70.05 ± 15.96	68.61 ± 17.14	0.451#
Body mass index	25.45 ± 4.17	26.01 ± 5.65	25.43 ± 4.11	26.03 ± 4.92	25.86 ± 4.17	0.915#
SBP (mmHg)	138.25 ± 19.87	141.94 ± 22.66	141.49 ± 21.90	138.48 ± 22.54	152.76 ± 28.80	0.082#
DBP (mmHg)	79.24 ± 12.37	79.92 ± 11.04	80.76 ± 12.60	79.70 ± 13.97	85.12 ± 18.14	0.418#
Comorbidity						
Hypertension	119 (32.3%)	51 (36.2%)	72 (33.0%)	35 (36.1%)	6 (35.3%)	0.910
lipidemia	100 (27.2%)	38 (27.0%)	75 (34.4%)	35 (36.1%)	9 (52.9%)	0.044
Ischemic heart disease	74 (20.1%)	34 (24.1%)	47 (21.6%)	19 (19.6%)	1 (5.9%)	0.501
Heart failure	31 (8.4%)	9 (6.4%)	26 (11.9%)	12 (12.4%)	1 (5.9%)	0.31
COPD	10 (2.7%)	2 (1.4%)	2 (0.9%)	0 (0.0%)	0 (0.0%)	0.372
Stroke	48 (13.0%)	17 (12.1%)	24 (11.0%)	11 (11.3%)	1 (5.9%)	0.937
Diabetic neuropathy	23 (6.2%)	17 (12.1%)	21 (9.6%)	9 (9.3%)	2 (11.8%)	0.185
Laboratory test						
HbA1c (%)	7.72 ± 1.92	7.99 ± 1.97	8.24 ± 1.79	8.54 ± 2.36	7.78 ± 1.97	<0.001
Last Glu AC (mg/dL)	144.12 ± 57.86	148.18 ± 53.03	150.22 ± 54.92	159.10 ± 71.30	123.53 ± 38.11	0.039
Triglyceride (mg/dL)	146.60 ± 118.68	150.05 ± 86.88	156.59 ± 153.53	170.53 ± 194.11	140.65 ± 69.66	0.776
Total Cholesterol (mg/dL)	171.83 ± 36.93	170.57 ± 41.43	169.39 ± 40.84	171.63 ± 40.85	177.59 ± 34.86	0.676
LDL Cholesterol (mg/dL)	101.95 ± 30.79	103.56 ± 36.36	98.59 ± 31.45	103.44 ± 33.64	115.35 ± 38.22	0.398
HDL Cholesterol (mg/dL)	48.18 ± 13.97	46.12 ± 11.95	46.82 ± 13.57	44.25 ± 11.60	50.82 ± 12.79	0.083
Uric Acid (mg/dL)	5.56 ± 1.46	5.64 ± 1.43	5.70 ± 1.65	6.03 ± 1.63	6.08 ± 1.38	0.118
Hemoglobin (g/dL)	13.28 ± 1.66	13.35 ± 1.75	12.91 ± 1.87	13.08 ± 1.77	11.41 ± 2.13	0.001
Albumin (g/dL)	3.91 ± 0.55	3.90 ± 0.58	3.85 ± 0.64	3.87 ± 0.59	3.82 ± 0.63	0.895
Result						
CKD event	5 (1.4%)	5 (3.5%)	11 (5.0%)	7 (7.2%)	1 (5.9%)	0.012
Time to CKD (years)	2.19 ± 1.65	2.05 ± 1.58	1.58 ± 1.38	2.19 ± 1.53	1.47 ± 1.15	<0.001

#: Testing by Fisher’s exact test, Wilcoxon test, or Kruskal–Wallis test, respectively. Significance level of *p* < 0.05 throughout the analysis. SBP = Systolic blood pressure, DBP = Diastolic blood pressure, COPD = chronic obstructive pulmonary disease, HbA1c = Glycated hemoglobin, Glu AC = Glucose before a meal, LDL = Low-density lipoprotein, HDL = High-density lipoprotein, CKD = chronic kidney disease.

**Table 5:** The risk of progression to CKD before the end of the study among patients without CKD initially.

	Crude-HR (95% CI)	*p*-Value	Adj-HR (95% CI)	*p*-Value #
Initial DR grade		0.004		0.012
No DR	1.00		1.00	
Mild NPDR	3.46 (0.92–12.98)	0.066	3.38 (0.90–12.71)	0.072
Moderate NPDR	8.75 (2.74–27.92)	<0.001	7.88 (2.43–25.57)	0.001
Severe NPDR	5.73 (1.64–20.04)	0.006	5.08 (1.35–19.04)	0.016
PDR	14.21 (1.55–130.67)	0.019	11.43 (1.22–107.12)	0.033
Gender		0.802		0.835
Female	1.00		1.00	
Male	0.91 (0.42–1.97)	0.802	0.92 (0.42–2.00)	0.835
age	0.79 (0.57–1.09)	0.148	0.69 (0.48–1.00)	0.047
height	0.80 (0.54–1.18)	0.263	0.69 (0.46–1.04)	0.077
weight	0.92 (0.65–1.30)	0.646	0.56 (0.32–0.99)	0.046
Body mass index	1.03 (0.71–1.49)	0.866	0.98 (0.67–1.42)	0.913
SBP	1.15 (0.81–1.64)	0.438	1.14 (0.81–1.60)	0.463
DBP	1.13 (0.79–1.62)	0.505	1.02 (0.70–1.48)	0.937
Comorbidity				
Hypertension	1.51 (0.71–3.22)	0.286	1.42 (0.65–3.12)	0.381
lipidemia	1.12 (0.50–2.52)	0.790	1.18 (0.51–2.70)	0.699
Ischemic heart disease	0.91 (0.36–2.33)	0.847	1.01 (0.39–2.58)	0.987
Stroke	0.48 (0.11–2.11)	0.332	0.73 (0.16–3.23)	0.674
Diabetic neuropathy	0.83 (0.24–2.88)	0.772	0.66 (0.16–2.72)	0.563
Heart failure	3.45 (1.43–8.29)	0.006	3.47 (1.43–8.40)	0.006
Laboratory test				
HbA1c	1.29 (0.97–1.71)	0.079	1.23 (0.88–1.73)	0.233
Glu AC	1.03 (0.75–1.42)	0.852	0.96 (0.67–1.38)	0.832
triglyceride	1.04 (0.79–1.38)	0.765	1.04 (0.80–1.37)	0.754
total Cholesterol	1.75 (1.17–2.59)	0.006	1.68 (1.10–2.54)	0.015
LDL Cholesterol	1.92 (1.32–2.78)	0.001	1.85 (1.24–2.77)	0.003
HDL Cholesterol	1.03 (0.71–1.49)	0.886	0.98 (0.66–1.45)	0.921
Hemoglobin	0.67 (0.45–1.00)	0.053	0.67 (0.45–1.00)	0.053

#: All results of Adj-HR were adjusted by sex, Age, BMI, eGFR. Significance level of *p* < 0.05 throughout the analysis. DR = diabetic retinopathy, NPDR = nonproliferative diabetic retinopathy, PDR = proliferative diabetic retinopathy, SBP = Systolic blood pressure, DBP= Diastolic blood pressure, HbA1c = Glycated hemoglobin, Glu AC = Glucose before a meal, LDL = Low-density lipoprotein, HDL = High-density lipoprotein.

## Data Availability

The data presented in this study are available on request from the corresponding author.

## Supplementary Materials

The following are available online at https://www.mdpi.com/2077-0383/10/1/3/s1, Figure S1. Kaplan–Meier survival curve, comparison of each CKD stages, showed worse baseline eGFR was related to the incidence of ESRD. Figure S2. Kaplan–Meier survival curve in all enrolled participants, comparison of each DR grade, showed DR grade was related to the incidence of ESRD. Figure S3. Kaplan–Meier survival curve in patient without CKD, comparison of each DR grade, showed DR grade was related to the progression to CKD.
