# Supplementary material for: The Severity of Diabetic Retinopathy Is an Independent Factor for the Progression of Diabetic Nephropathy
Source: J Clin Med. 2020 Dec 22;10(1):3. doi: 10.3390/jcm10010003 (PMC7792601; doi:10.3390/jcm10010003)
Supplement: Supplementary file 1 [file jcm-10-00003-s001.pdf]

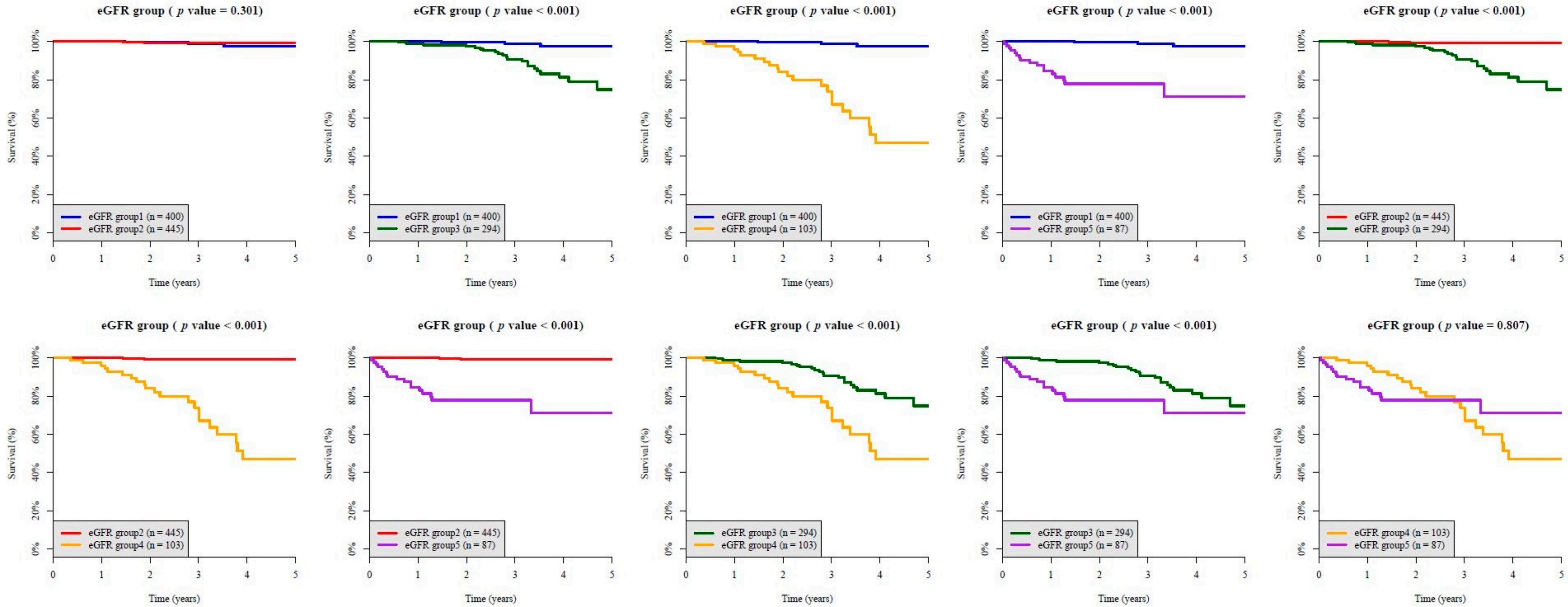

Figure 1. Kaplan–Meier survival curve, comparison of each CKD stages, showed worse baseline eGFR was related to the incidence of ESRD.

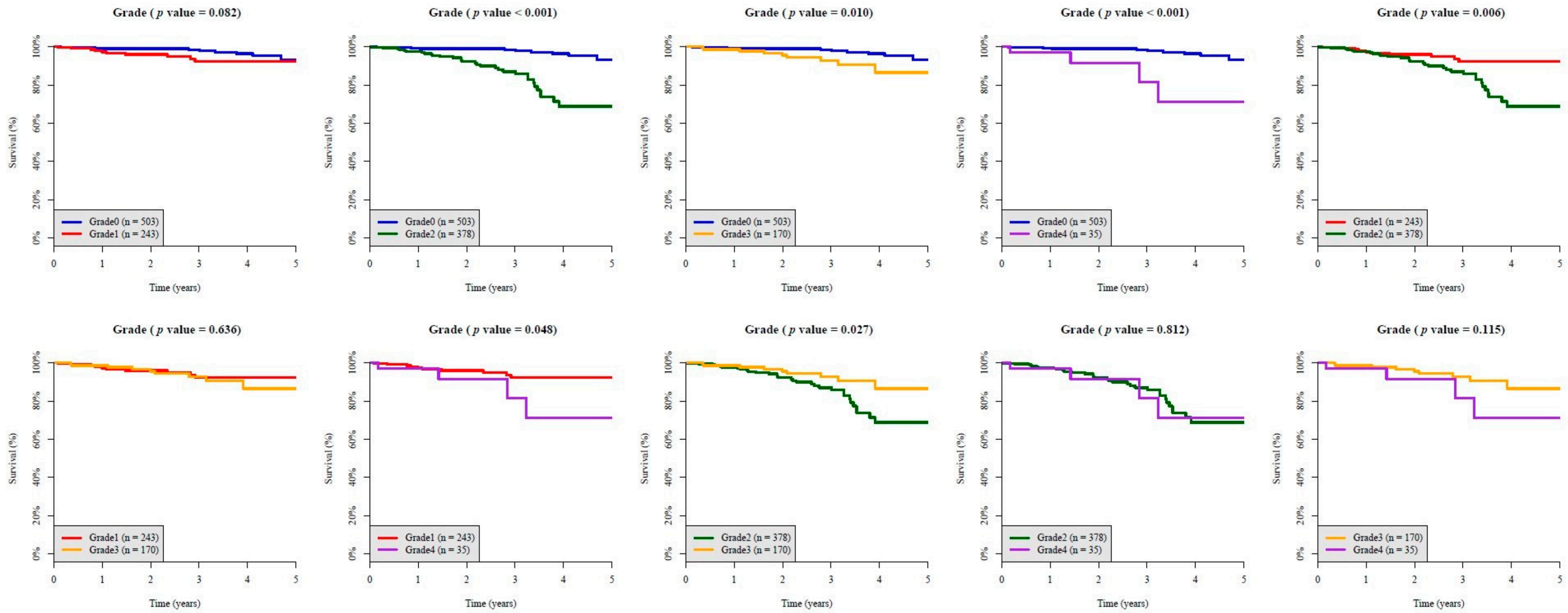

Figure 2. Kaplan–Meier survival curve in all enrolled participants, comparison of each DR grade, showed DR grade was related to the incidence of ESRD.

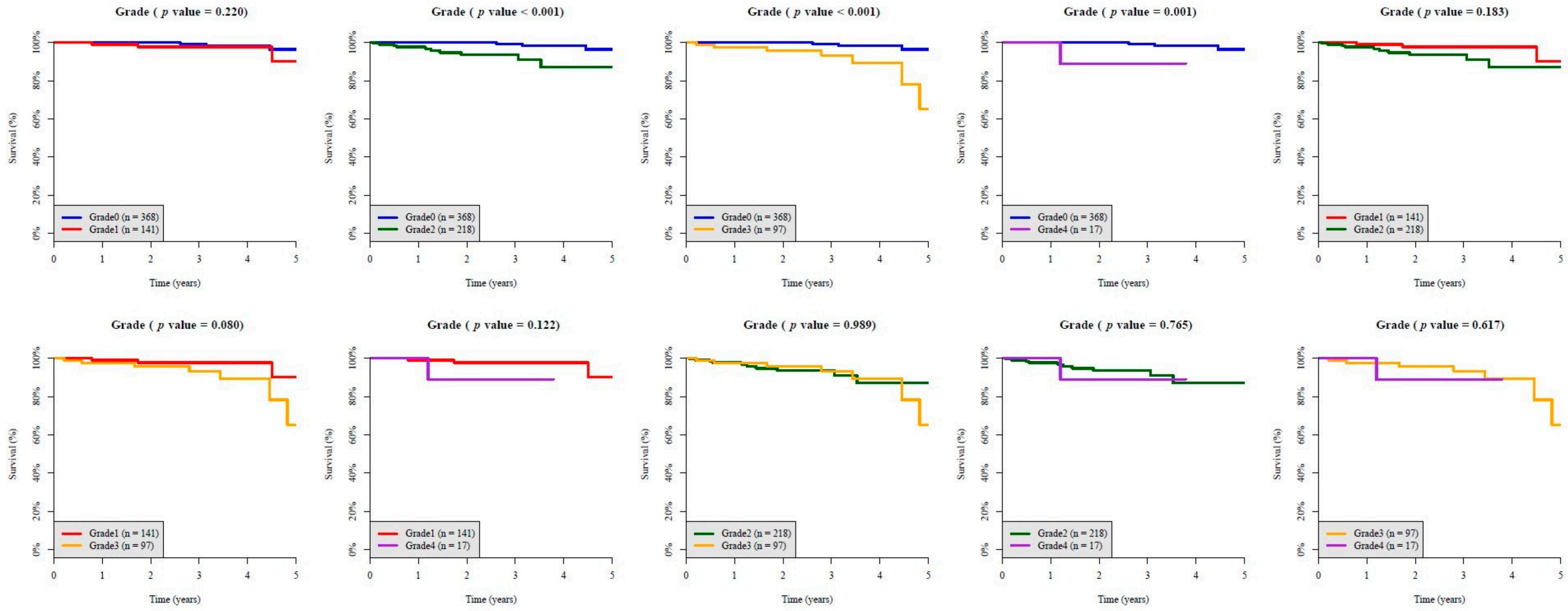

Figure 3. Kaplan–Meier survival curve in patient without CKD, comparison of each DR grade, showed DR grade was related to the progression to CKD.
